# Meta-Analysis of the Copper, Zinc, and Cadmium Absorption Capacities of Aquatic Plants in Heavy Metal-Polluted Water

**DOI:** 10.3390/ijerph121214959

**Published:** 2015-11-26

**Authors:** Jing Li, Haixin Yu, Yaning Luan

**Affiliations:** College of Forestry, Beijing Forestry University, Beijing 100083, China; lijing9411@bjfu.edu.cn (J.L.); yuhaixin@bjfu.edu.cn (H.Y.)

**Keywords:** phytoremediation, meta-analysis, heavy metals, aquatic plants, cadmium metal absorption, copper metal absorption, zinc metal absorption

## Abstract

The use of aquatic plants for phytoremediation is an important method for restoring polluted ecosystems. We sought to analyze the capacity of different aquatic plant species to absorb heavy metals and to summarize available relevant scientific data on this topic. We present a meta-analysis of Cu, Zn, and Cd absorption capacities of aquatic plants to provide a scientific basis for the selection of aquatic plants suitable for remediation of heavy-metal pollution. Plants from the Gramineae, Pontederiaceae, Ceratophyllaceae, Typhaceae and Haloragaceae showed relatively strong abilities to absorb these metals. The ability of a particular plant species to absorb a given metal was strongly correlated with its ability to absorb the other metals. However, the absorption abilities varied with the plant organ, with the following trend: roots > stems > leaves. The pH of the water and the life habits of aquatic plants (submerged and emerged) also affect the plant’s ability to absorb elements. Acidic water aids the uptake of heavy metals by plants. The correlation observed between element concentrations in plants with different aquatic life habits suggested that the enrichment mechanism is related to the surface area of the plant exposed to water. We argue that this meta-analysis would aid the selection of aquatic plants suitable for heavy-metal absorption from polluted waters.

## 1. Introduction

Rapid large-scale industrialization and the production of a variety of chemical compounds have caused severe environmental pollution, especially heavy metal pollution. Recent studies have shown that aquatic heavy metal pollution is an important aspect of ecological degradation [1]. The persistence of heavy metals in the ecosystem and their bioaccumulation as they move through food chains causes them to present a substantial health danger to humans [2]. Pollution with copper (Cu), zinc (Zn) and cadmium (Cd) is particularly serious because these are frequent heavy metal contaminants present in water, and reach toxic concentrations in aquatic foodstuffs through food-chain biomagnification. Hence, phytoremediation, or the use of plants to remove or break down toxic contaminants in the environment, is currently of great research interest. Plants that can absorb and store heavy metals can be used to remove those pollutants from an ecosystem. Aquatic plants can accumulate elements through their roots, stems, and leaves [3]. Various species show different capacities for metal uptake, and the use of these species for bioremediation has numerous economic and ecological benefits, including low cost, high efficiency, energy savings, and prevention of secondary pollution [4].

The efficiency of aquatic plants for phytoremediation has been documented by many traditional narrative reviews [5,6]. Many studies have summarized existing evidence, but most have not used a systematic or statistical method to synthesize and evaluate the information collected. On the other hand, a meta-analysis is a research method that uses statistical methods to combine results from different studies. This study used meta-analysis to examine the capacity of various hydrophytes to absorb Cu, Zn, and Cd. The goal of these analyses was to provide a scientific basis for selecting suitable hydrophytes for phytoremediation in cases of heavy metal pollution.

The objectives of this study were: (1) to analyze the capacity of different plant species to absorb Cu, Zn, and Cd; (2) to examine whether the capacity to absorb one of these metals is correlated with the ability to absorb the others; and (3) to summarize, through a systematic meta-analysis, all available scientific data on the absorption of Cu, Zn, and Cd by aquatic plants.

## 2. Materials and Methods

### 2.1. Documentation Indexing

An extensive analysis of the available literature (from 1995 up to 2015) was used to identify studies that calculated the capacity of aquatic plants to absorb heavy metals. All literature surveyed for the meta-analysis in this paper was from Elsevier, Wiley, or Springer. Keywords included phytoremediation, aquatic plant, wetland plant, heavy metal, bioconcentration factor (BCF), copper (Cu), zinc (Zn), and cadmium (Cd). The studies included in this analysis met the following criteria: (1) The aquatic plant being studied absorbs Cu, Zn, and/or Cd and (2) it was possible to calculate the effect size (ES) from the data available on metal absorption by the aquatic plant.

The exclusion criteria included multiple publications and articles for which the BCF could not be calculated for statistical analysis. Fifteen articles examining 51 species belonging to 18 families (Table 1) were included in the analysis and conformed to the requirements.

### 2.2. Statistical Analysis

For integration analysis, each research study was required to be independent; therefore, it was assumed that studies in different locations with different pH values, heavy metal pollution conditions, or aquatic plant life habits were independent.

To eliminate differences caused by growing conditions (e.g. field or pots), species, initial metals concentration, and other sources of variation, the BCF has been calculated as a response ratio (R) to be used as a metric of the ES in the meta-analysis. This ratio has been calculated as log_e_R = log_e_(X_d_/X_c_), where X_d_ and X_c_ were the mean values for the experiment and control groups [22]. In detail, X_d_ was the concentration of chemical in the biota, whereas X_c_ was the concentration of metal in the water [23,24]. The ratios were log-transformed before analysis to compress observed variability without changing the relationship between data points [23]. A value of log_e_R = 0 implies that the aquatic plants used for phytoremediation had no effect. The variance of log_e_R for each study was calculated using the inverse of the pooled variance [22]. We calculated the mean effect values and generated 95% confidence intervals (CIs) by using the random-effects model in MetaWin 2.1 [25]. For bootstrapping we used 4999 iterations. The difference between the control and experimental groups were considered significant if the 95% CI did not overlap with zero. The use of more than one observation within a study may have overrepresented an effect from studies with a large number of observations.

SPSS version 15.0 software (SPSS Inc., Chicago, IL, USA) was used to estimate correlations among a plant’s BCFs for all pairwise combinations of the three metals under consideration. Each study was counted as a separate data point among plant species. The threshold for significance was set at α = 0.05. SPSS version 15.0 was used to calculate means and standard errors for BCFs for different organs of the plants.

**Table 1:** Additional information extracted from selected studies to evaluate the effect of aquatic plants on adsorption of Cu, Zn, and Cd.

	Experimental Site	pH	Family	Heavy Metal Concentration (mg·L^−1^)			Study
				Cu	Zn	Cd	References
1	Shenzhen Special Economic Zone, China	3.65	Rhizophoraceae	-	7.26	0.77	[7]
2	Part of Unnao city, U. P., India	7.30	Typhaceae, Cyperaceae	2.61	2.79	0.96	[8]
3	Zhejiang Province, China	-	Araceae, Cyperaceae, Gramineae, Iridaceae, Juncaceae, Lythraceae, Pontederiaceae, Typhaceae	0.05	7.72	-	[9]
4	West Bengal, India	8.75	Convolvulaceae, Compositae, Marsileaceae	0.09	0.15	0.07	[10]
5	Southern Assam, India	-	Amaranthaceae, Araceae, Athyriaceae, Chenopodiaceae, Compositae, Convolvulaceae, Cyperaceae, Euphorbiaceae, Labtatae, Leguminosae, Onagraceae, Pontederiaceae, Solanaceae, Umbelliferae	-	1.48	-	[11]
6	Northeast of Nantes, France	-	Juncaceae, Typhaceae	0.25	2.00	0.10	[12]
7	Northwest of Lake Taihu, China	7.62	Gramineae, Onagraceae	0.74	2.59	0.12	[13]
8	River Olobok, Poland	7.00	Hydrocharitaceae, Potamogetonaceae	1.91	-	0.22	[14]
	River Pilawa, Poland	6.60	Hydrocharitaceae, Potamogetonaceae	4.87	-	0.75	[14]
9	Southern Jiangsu Province, China	7.30	Ceratophyllaceae	0.89	9.10	0.12	[15]
10	Olesno, Poland	-	Typhaceae	7.26	5.10	-	[16]
11	South Bohemia, CzechRepublic	-	Gramineae, Typhaceae	0.68	4.96	0.02	[17]
12	Šalek Valley, Slovenia	12.0	Najadaceae, Potamogetonaceae	1.20	2.00	-	[18]
13	Lucknow, U. P., India	6.48	Asclepiadaceae, Chenopodiaceae, Compositae, Cyperaceae, Malvaceae, Solanaceae, Euphorbiaceae	4.25	-	0.02	[19]
14	Barra do Pira´s, Brazil	6.80	Leguminosae, Araceae, Pontederiaceae	1.89	3.38	0.26	[20]
15	Sohag City, Egypt	7.6	Ceratophyllaceae, Pontederiaceae, Haloragidaceae, Gramineae, Typhaceae	0.03	0.11	0.01	[21]

### 2.3. Publication Bias

Publication bias occurs when researchers, reviewers, and editors publish papers whose results are influenced by factors external to the scientific question being addressed, for example, the tendency for studies to be published only when an effect is positive or considered significant based on peer review. Bias may also occur against studies not written in English or those containing results unfavorable to a study’s sponsor. The statistical (Begg’s and Egger’s test), graphical (funnel plot) and “trim-and-fill” methods were used to eliminate and correct publication bias. Outliers having large standard errors (SE) or low statistical effects were deleted until the funnel plot became symmetrical. The omitted studies were replaced with their “missing” counterpart studies, and the center of the plot was re-estimated [26]. This method evaluates the extent to which the average estimate of treatment effect changes if studies are missing because of publication bias [27].

## 3. Results and Discussion

### 3.1. BCF for Copper, Zinc, and Cadmium

**Figure 1:**
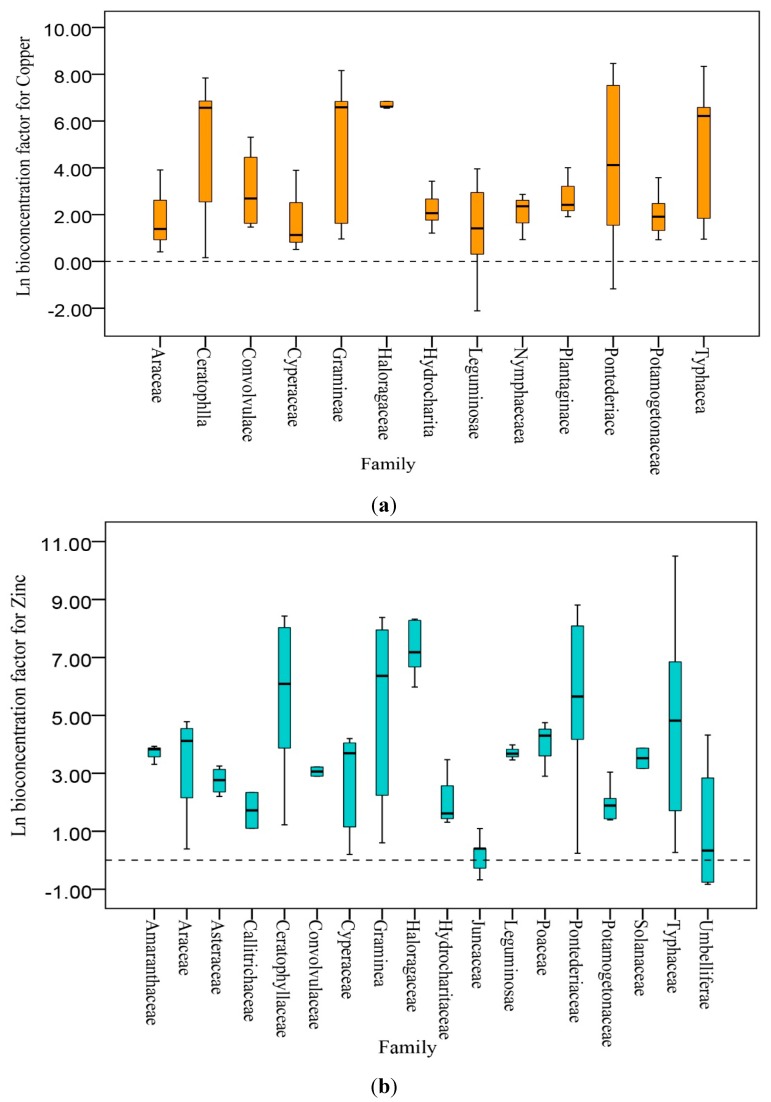

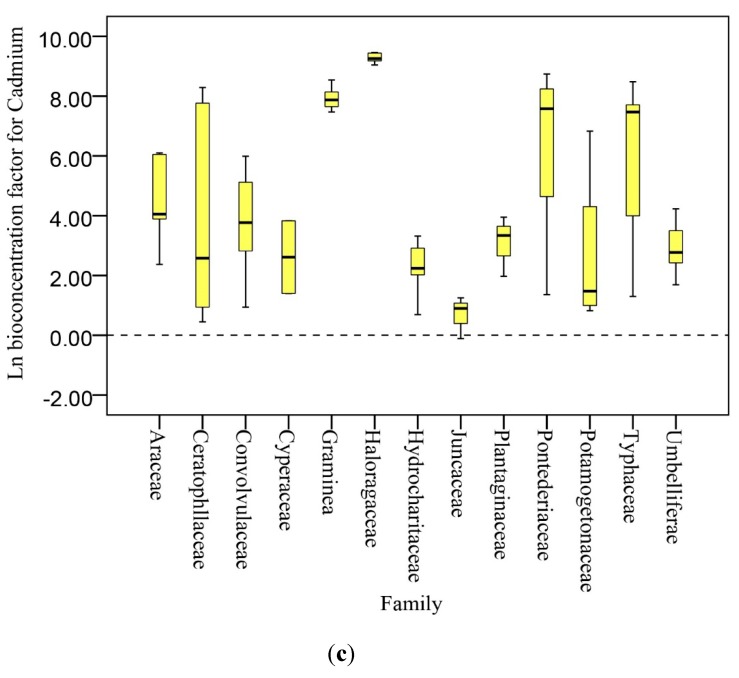
(**a**) Bioconcentration of copper by aquatic plants belonging to 13 families. (**b**) Bioconcentration of zinc by aquatic plants belonging to 18 families. (**c**) Bioconcentration of cadmium by aquatic plants belonging to 13 families. The Ln BCF is noted for each study. The whiskers represent the range, the hinges represent the inter-quartile range (IQR), and the middle line represents the median.

The study indicated that a total of 69 aquatic plant species, belonging to as many as 19 different families, were able to grow, and tolerate high levels of Cu, Zn, and Cd in water. The mean (μ) BCF for Cu was similar across all 13 plant families (*n* = 110, median = 2.87, min = −2.12, max = 8.46; Figure 1a). A higher BCF for Cu was observed in plants of the family Gramineae (*n* = 13, median = 6.59, min = 0.96, max = 8.16), of which the most frequently studied species was *Phragmites australis* (Cav.) Trin. ex Steud; Pontederiaceae (n = 23, median = 6.62, min = −1.18, max = 2.82), of which the most frequently studied species was *Eichhornia crassipes* (Mart.) Solms; Typhaceae (*n* = 11, median = 6.21, min = 0.95, max = 8.34), of which the most frequently studied species was *Typha angustifolia* L.; Ceratophyllaceae(*n* = 13, median = 6.57, min = 0.16, max = 7.84), of which the most frequently studied species was *Ceratophyllum demersum* L.; and Haloragaceae (*n* = 6, median = 6.62, min = 6.31, max = 6.84), of which the most frequently studied species was *Myriophyllum spicatum* L.

These results are similar to those of a previous study [28]. As a cofactor for enzymes involved in both respiration and photosynthesis, Cu is a vital nutrient for plants. It also plays important roles in plant growth and development. However, excessive concentrations of this metal are considered highly toxic [29,30]. A previous study showed that plants and other organisms regulate intracellular copper levels by regulating copper uptake and by reducing intracellular free copper concentrations through metallochaperones [31], which are soluble Cu-binding proteins that deliver Cu to sites where it is needed within the cell [32].

The mean (μ) BCF for Zn was similar for all 18 plant families (n = 112, median = 3.87, min = −0.83, max = 10.5; Figure 1b). The BCF of Zn exceeded 1 for most of the aquatic plants, indicating their high tolerance for Zn. As with Cu, the following families all had a high ability to absorb Zn: Pontederiaceae (*n* = 17, median = 5.65, min = 0.24, max = 8.81), of which the most frequently studied species was *E. crassipes*); Ceratophyllaceae (*n* = 13, median = 6.09, min = 1.22, max = 8.43),of which the most frequently studied species was *C. demersum*; Typhaceae (n = 14, median = 4.82, min = 0.27, max = 10.5), of which the most frequently studied species was *T. angustifolia* L.; Gramineae (*n* = 13, median = 6.36, min = 0.60, max = 8.38), of which the most frequently studied species was *P. australis*; and Haloragaceae (*n* = 5, median = 7.18, min = 5.98, max = 8.32), of which the most frequently studied species was *M. spicatum*.

Zinc is one of the necessary trace elements in plants, and it plays an important role in plant growth development. Zinc is found in some enzymes, such as polyphenol oxidase, ascorbic acid oxidase, and cytochrome oxidase [33,34,35]. According to a previous study [36], when plants absorb the heavy metal, zinc is transformed from the insoluble to the soluble Zn^2+^ state. Simultaneously, it also activates the insoluble state in the water, which could increase the ability of aquatic plants to absorb zinc.

The mean (μ) BCF for Cd was similar across all 13 plant families (*n* = 84, median = 4, min = −0.11, max = 9.46; Figure 1c), with the exceptions of the Gramineae, Pontederiaceae, Typhaceae and Haloragaceae. A higher BCF for Cd was observed in plants of the family Gramineae (n = 7, median = 7.78, min = 7.47, max = 8.54), of which the most frequently studied species was *P. australis*; Pontederiaceae (n = 13, median = 7.54, min = 1.36, max = 8.74), of which the most frequently studied species was *E. crassipes*; Typhaceae (of which the most frequently studied species was *T. angustifolia* L. (*n* = 8, median = 7.47, min = 1.30, max = 8.48); and Haloragaceae (*n* = 5, median = 9.26, min = 9.04, max = 9.46), of which the most frequently studied species was *M. spicatum*.

Cd is rather mobile in soils and thus readily available for plants, although the uptake mechanisms are not well known [28]. Plants of both the Gramineae and Pontederiaceae show high Cd-tolerance [37], possibly due of a defense strategy based on increased antioxidant enzyme activity [38].

### 3.2. Relationship among Copper, Zinc, and Cadmium

**Figure 2:**
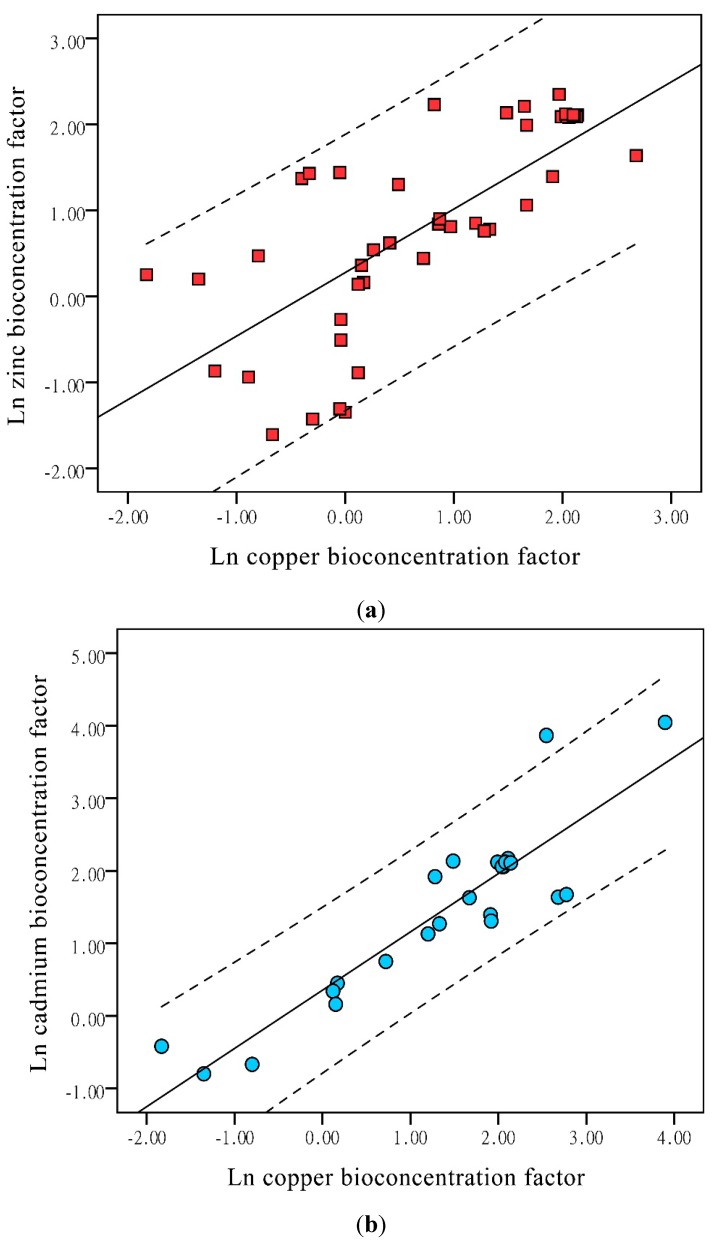

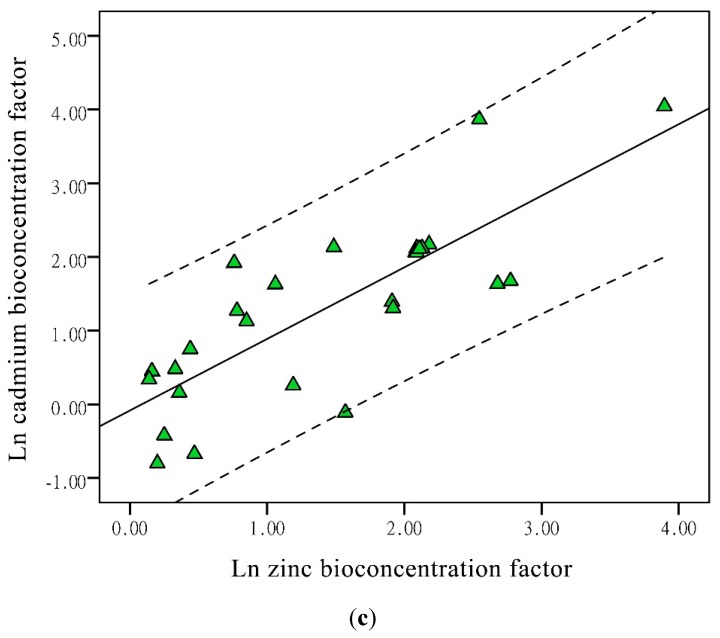
Correlations of bioconcentration factors between pairs of heavy metals (**a**) copper and zinc; (**b**) copper and cadmium; (**c**) zinc and cadmium.

BCFs for Cu and Zn (*r* = 0.74, *p* < 0.01; Figure 2a) were significantly correlated. BCFs for Cu and Cd (*r* = 0.903, *p* < 0.01; Figure 2b) were highly correlated, similar to previous study [39]. As with the other two pairs of metals, the BCFs for Zn and Cd (*r* = 0.803, *p* < 0.01; Figure 2c) were significantly correlated.

This pattern suggests that the process of concentration of Cu and Zn in aquatic plants may be cooperative. According to a previous study [40], ion transportation across the membrane is the primary approach by which metals enter a plant cell, and ion channels are the most important regulatory mechanism. This mechanisms of toxicity suggested that Cu and Zn are transported together. The BCFs for Cu and Cd may be more closely correlated than those for Cu and Zn, because Cu and Zn have different mechanisms of toxicity. According to a previous study [41], Cu is a redox-active metal that can produce reactive oxygen species (ROS) directly via Fenton and Haber-Weiss reactions. However, cadmium does not participate in redox reactions and causes oxidative stress via indirect mechanisms such as interactions with enzymes of the antioxidative defense system [42]. Although they both induce mitogen activated protein kinase (MAPKs), Cd and Cu use distinct signaling pathways depending on the type of ROS generated [43]. The observed correlation between BCFs for Zn and those for Cd agrees broadly, but not exactly, according to the results of a previous study [12], which may be explained by the transporters and channels through which Cd and Zn are moved [44]. The discrepancy relative to the previous study may be explained by other factors in water that affect heavy metal uptake, such as pH [45] or other ions (e.g., Fe, Ca) in plants [46].

### 3.3. Influencing Factors

#### 3.3.1. Plant Organs

**Figure 3:**
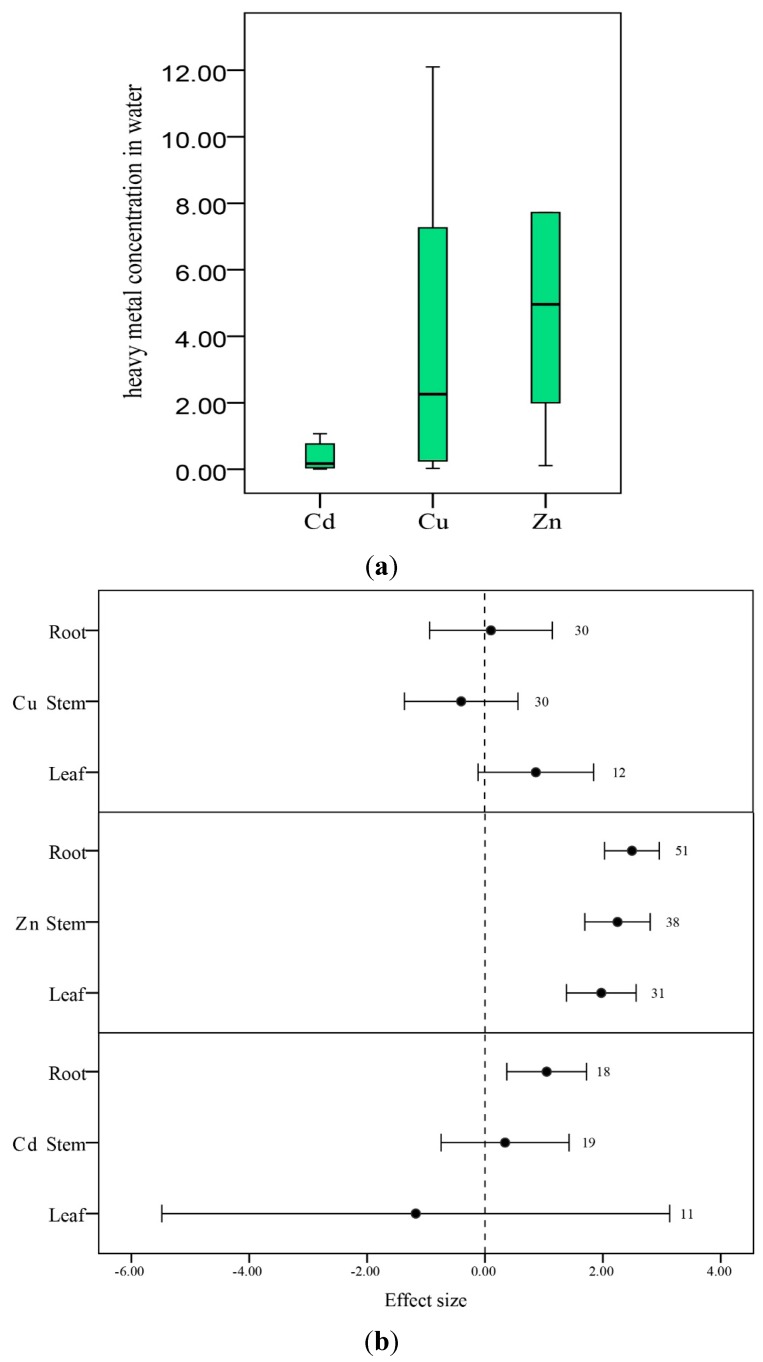
(**a**) The Cu, Zn and Cd concentration in water (mg·L^−1^). The whiskers represent the range, the hinges represent the inter-quartile range (IQR), and the middle line represents the median. (**b**) The mean bioconcentration ratio values of heavy metals Zn, Cu, and Zn concentration on aquatic plant organs categorized by root, stem, and leaves. Error bars represent 95% bootstrapped confidence intervals (CIs). The effect of heavy metal concentration ability in aquatic plants was considered significant if the 95% CI of the effect size did not overlap with zero. The number of observations for each category is shown next to the error bars.

The pattern of accumulation of the three metals BCFs for Zn (Median = 4.96 mg·L^−1^) tended to be higher than that for Cu (Median = 2.26 mg·L^−1^), which tended to be higher than that for Cd (Median = 0.17 mg·L^−1^) (Figure 3(a)). This occurs because Cu and Zn are essential for plant growth and metabolism [35].

The distribution of heavy metals in different plant parts varied between metal species. As seen in Figure 3b, for cadmium and zinc, the roots accumulated significantly higher concentrations of the metals, whereas the leaves had lower concentrations of metals than did the stems. The roots have been known as good absorptive sponge for heavy metals in soil and water. Similar findings have been reported by various authors for heavy metal uptake in water [8,9,47,48]. A possible reason is that the roots are the primary site of metal uptake. However, in aquatic plants, the stem appears to have a greater ability to absorb heavy metals than it does in terrestrial plants [49]. Metals absorbed or adsorbed by roots are often bound by cell wall material or other macromolecules to prevent them from being translocated to sensitive plant parts [50]. The concentration of copper was higher in leaves, potentially because the capacity of roots was became exhausted due to a high concentration of Cu in wastewater [22].

#### 3.3.2. pH

**Figure 4:**
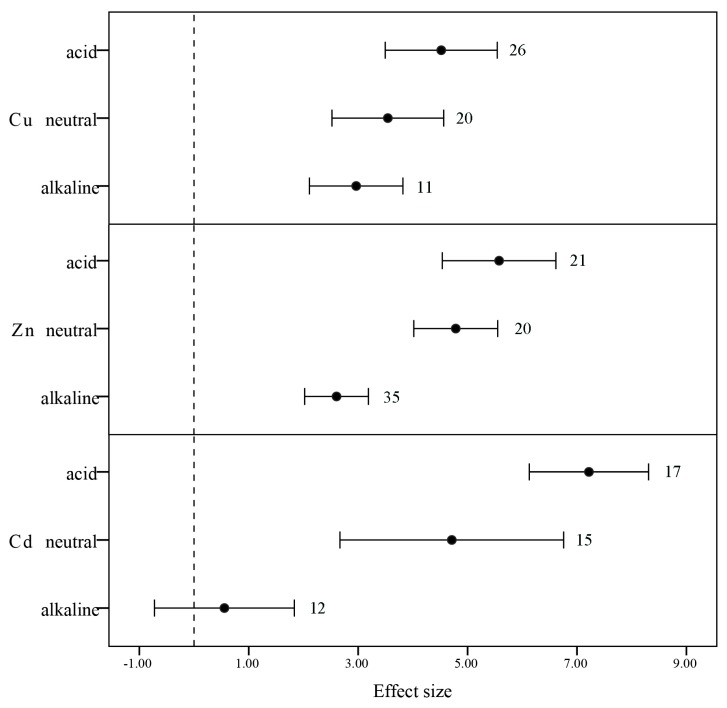
The mean BCFs values of heavy metals Zn, Cu, and Zn concentration on aquatic plant categorized by pH: acidic (5.5–6.5), neutral (6.5–7.5), and alkaline (7.5–8.5). Error bars represent 95% bootstrapped confidence intervals (CIs). The effect of heavy metal concentration ability in aquatic plants was considered significant if the 95% CI of the effect size did not overlap with zero. The number of observations for each category is shown next to the error bars.

The pH value is one of the most important factors controlling metal availability [51]. Each body of water has an individual pattern of physical and chemical characteristics that are determined largely by climate, geomorphological, geochemical, and human activities. In one instance, mining activity increased the water pH to 12 [18]. Our results indicated that slightly acidic water, with a pH of 5.6–6.5, aids the uptake of heavy metals by plants. According to Zeng *et al.* [52], the accumulation potential of the investigated plants at different pH consistently followed the pattern: acidic > neutral > alkaline (Figure 4). The concentrations of heavy metals in water and soil are usually negatively correlated with pH [34]. Generally, the efficiency of heavy metal ion adsorption will have an optimal pH range. When the pH value is high, the ions exist by insoluble oxide, hydroxide statement, which the plants hardly absorb heavy metals. When the pH value is low, organic acids and H+ competition will lead to ions replaced by H_3_O^+^, which mobilize heavy metal ions from sediment and increase the possibility of aquatic plants of absorption heavy metals [53].

#### 3.3.3. Submerged and Emerged Plant Species

**Figure 5:**
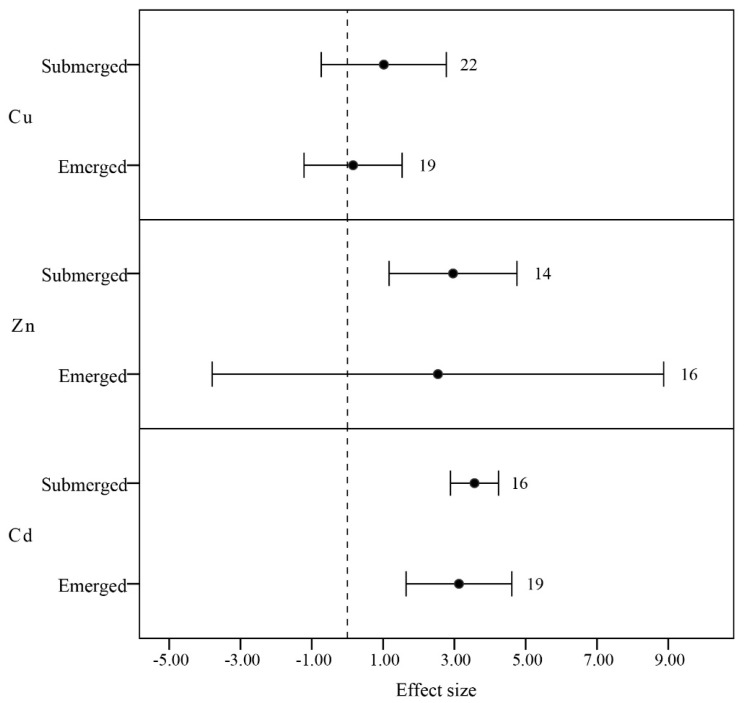
Mean BCF values of concentration of heavy metals Zn, Cu, and Cd on aquatic plant categorized by life habits: emerged and submerged. Error bars represent 95% bootstrapped confidence intervals (CIs). The effect of heavy metal concentration ability in aquatic plants was considered significant if the 95% CI of the effect size did not overlap with zero. The number of observations for each category is shown next to the error bars.

According to Albers and Camardese [54], submerged species generally accumulate relatively higher concentrations of the heavy metals copper, zinc, and cadmium, as compared to emerged species (Figure 5). According to Yurukova’s study [55], this is probably because some emerged plants’ roots degrade or disappear, such as *Ceratophyllum demersum* L., which do not have roots but develop modified leaves with a root like appearance, and because their waxy coat inhibits absorption by epidermal cells. The enrichment mechanism may also be related to the surface area of the plant exposed to water, in that a higher surface area: volume ratio would enable higher uptake of heavy metals [47].

#### 3.3.4. Soil, Water, and Plants

**Figure 6:**
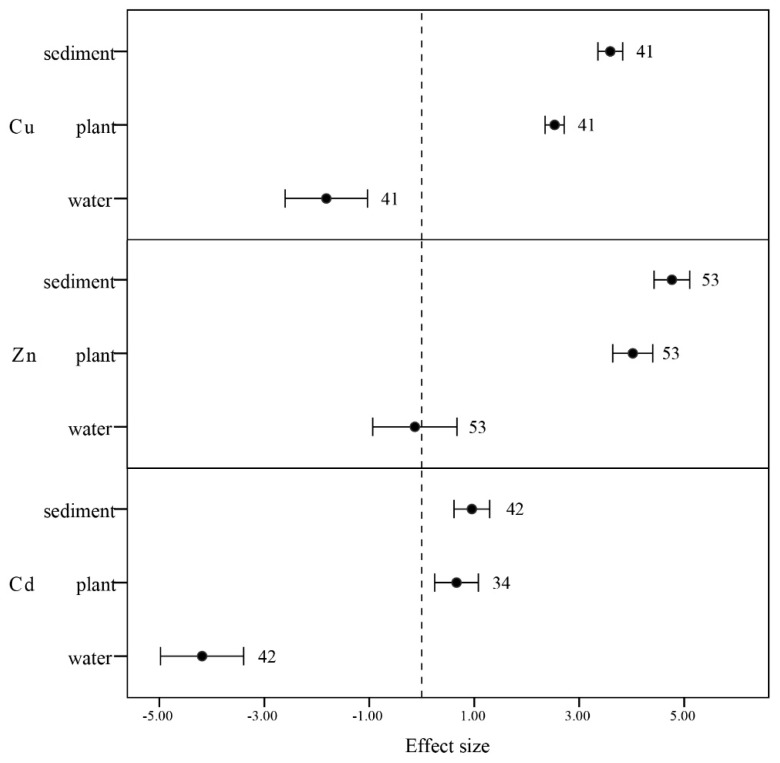
Mean BCF values of concentration on aquatic plants, water, and sediments categorized by Zn, Cu, and Cd. Error bars represent 95% bootstrapped confidence intervals (CIs). The effect of heavy metal concentration ability in aquatic plants was considered significant if the 95% CI of the effect size did not overlap with zero. The number of observations for each category is shown next to the error bars.

We used the Araceae, Ceratophyllaceae, Convolvulaceae, Cyperaceae, Gramineae, Haloragaceae, Pontederiaceae, and Solanaceae to evaluate the relationship among soil, water and plants. Heavy metal concentrations tended to be the lowest in water and the highest in sediments (Figure 6). In addition, the mean heavy metal concentrations in water, sediments, and plants possess the same trend: Zn > Cu > Cd, which reflects the biomonitoring potentialities of the examined plant species.

The concentration of metals in water was not particularly high, but the sediment and aquatic plants both accumulated high concentrations of metals. These results are in agreement with those of Sahu [48]. A likely explanation is that under continuous chronic exposure of the plant, heavy metals modify metabolic pathways by increasing the demand for essential nutrients [56]. After the growth of several generations of aquatic plant species in the polluted area, continuous exposure may lead to the selection of those species that are most tolerant of heavy metal accumulation [57]. However, the bioconcentration factors and heavy metals concentrations in water far smaller than heavy metal concentration in sediments indicating that the soil was very effective in binding heavy metals and the uptake by plants decreased with increasing input concentrations [58].

## 4. Conclusions

Many heavy metals reach toxic levels as their concentrations become magnified through the food chain. This has warranted a focus on methods for removing these pollutants from the environment. Phytoremediation of heavy metals is an eco-friendly and innovative method for removing these toxic metals. This study conducted a meta-analysis of previous research on the capacity of aquatic plants to absorb Cu, Zn, and Cd. Plants belonging to families such as Gramineae, Pontederiaceae, Ceratophyllaceae, Typhaceae, and Haloragaceae have a relatively high ability to absorb these metals. Uptake of one of these metals by a plant is generally highly correlated with the uptake of the others. Roots tend to absorb more metals than stems, which in turn absorb more metals than leaves. The pH was negatively correlated with the ability of the aquatic plant to absorb copper, zinc, and cadmium, suggesting that pH played an important role in heavy metal accumulation by plants. The correlation found between element concentrations in the different aquatic life habits—submerged and emerged—suggested that the enrichment mechanism is related to the surface area of the plant exposed to water, which would help in the selection of the suitable aquatic plants for absorption of heavy metals from polluted water.

Through the analysis of their concentration in soil, water, and aquatic plants, the observed high concentration of heavy metals in aquatic plants indicates that some species could accumulate high level of metals even when the concentration of metal in the water is not particularly high.

We still lack a full understanding of the mechanisms and pathways by which aquatic plants absorb and accumulate heavy metal ions. The capacity of aquatic plants to absorb heavy metals, the mechanisms by which such uptake occurs, and the effects of heavy metal accumulation in aquatic plants may be different from the capacity, mechanisms, and effects in non-aquatic plants. Further research should address the effects of accumulation as a function of growth phase, time of exposure, and changes in the external environment. Such research should allow us to further effectively apply our theory to practice in the field.
